# Intermittent rapamycin feeding recapitulates some effects of continuous treatment while maintaining lifespan extension

**DOI:** 10.1016/j.molmet.2024.101902

**Published:** 2024-02-13

**Authors:** Maarouf Baghdadi, Tobias Nespital, Carolina Monzó, Joris Deelen, Sebastian Grönke, Linda Partridge

**Affiliations:** 1 Max-Planck Institute for Biology of Ageing, Cologne, Germany; 2 Cluster of Excellence Cellular Stress Responses in Aging-associated Diseases (CECAD), Faculty of Mathematics and Natural Sciences, University of Cologne, Cologne, Germany; 3 Institute for Integrative Systems Biology, Spanish National Research Council, Catedràtic Agustín Escardino Benlloch, Paterna, Spain; 4 Institute of Healthy Ageing and Department of Genetics, Evolution and Environment, University College London, London, UK

**Keywords:** Rapamycin, Sex difference, Ageing, Metabolism, Cancer, Inflammation

## Abstract

**Objective:**

Rapamycin, a powerful geroprotective drug, can have detrimental effects when administered chronically. We determined whether intermittent treatment of mice can reduce negative effects while maintaining benefits of chronic treatment.

**Methods:**

From 6 months of age, male and female C3B6F1 hybrid mice were either continuously fed with 42 mg/kg rapamycin, or intermittently fed by alternating weekly feeding of 42 mg/kg rapamycin food with weekly control feeding. Survival of these mice compared to control animals was measured. Furthermore, longitudinal phenotyping including metabolic (body composition, GTT, ITT, indirect calorimetry) and fitness phenotypes (treadmil, rotarod, electrocardiography and open field) was performed. Organ specific pathology was assessed at 24 months of age.

**Results:**

Chronic rapamycin treatment induced glucose intolerance, which was partially ameliorated by intermittent treatment. Chronic and intermittent rapamycin treatments increased lifespan equally in males, while in females chronic treatment resulted in slightly higher survival. The two treatments had equivalent effects on testicular degeneration, heart fibrosis and liver lipidosis. In males, the two treatment regimes led to a similar increase in motor coordination, heart rate and Q-T interval, and reduction in spleen weight, while in females, they equally reduced BAT inflammation and spleen weight and maintained heart rate and Q-T interval. However, other health parameters, including age related pathologies, were better prevented by continuous treatment.

**Conclusions:**

Intermittent rapamycin treatment is effective in prolonging lifespan and reduces some side-effects of chronic treatment, but chronic treatment is more beneficial to healthspan.

## Highlights

• Intermittent rapamycin treatment extends lifespan to the same extent as chronic feeding in male but not female mice.
• Intermittent rapamycin feeding increases metabolic health compared to chronic feeding.
• Chronic rapamycin treatment is more efficient in reducing tumour growth and age-related inflammation.

## 1 Introduction

Human life expectancy has greatly increased in the past two centuries but healthy years have not kept up [1]. Advancing age is the predominant risk factor for most diseases [2], including the major chronic conditions [3], and there is considerable interest in improving health later in life to benefit both older people and the societies in which they live [4]. One geroscience approach is to elucidate the underlying biological mechanisms of ageing, with the aim of developing preventative therapies to simultaneously target the aetiology of multiple age-associated diseases [5]. A key development has been the design of a robust methodology to test the effect of pharmacological interventions on lifespan in mice in three independent research centres in the US [6]. Rapamycin, a disruptor of mammalian target of rapamycin complex 1 (mTORC1) [7], has emerged as a leading pharmacological intervention that increases lifespan and many parameters of health during ageing in multiple genetic strains of mice [8,9], even when started late in life and when given intermittently or transiently [[10], [11], [12], [13]]. mTOR inhibitors have also been successfully used in elderly humans to improve immune function [14]. Rapamycin-based compounds (also called rapalogs) are thus prime geroprotective candidates and have already been approved for more clinical trials in humans [15,16]. However, studies using chronic rapamycin treatment in mice have also reported negative consequences, namely an increased incidence of cataracts, testicular degeneration [17], glucose intolerance [9,18] and reduced blood T regulatory cells [19].

Intermittent administration of medications can achieve similar efficacy compared to continuous administration, while preventing some side effects of the treatment [[20], [21], [22], [23], [24], [25]]. Interestingly, previous studies on rapamycin have demonstrated clear efficacy of intermittent rapamycin treatment in extending lifespan in female mice [13,26], while partially rescuing some adverse effects of continuous treatment such as glucose intolerance and T-cell number [19]. This is probably because of avoidance of the inhibition of the TORC2 complex observed under chronic treatment [18,27]. Intermittent rapamycin treatment initiated in early life (i.e. at two months of age) can still achieve some of the health benefits observed under continuous rapamycin treatment, such as reduced spontaneous tumorigenesis [13]. Furthermore, rapamycin treatment starting later in life, i.e. at 20 months of age, can still extend mouse lifespan [28]. The translational potential of late-life rapamycin treatment thus encourages further exploration. Rapamycin is a particularly promising candidate for effective intermittent treatment, because it induces long-lasting effects after the drug has been cleared, in both *Drosophila* and mice [29,30].

In this study, we determined whether intermittent feeding of rapamycin, applied to both male and female mice, is sufficient to improve health parameters and extend lifespan, as previously described for chronic rapamycin treatment [9]. We also assessed whether adverse effects induced by continuous rapamycin treatment were reduced. Our study differed from previous work in two main respects: 1) we included both males and females and 2) we included continuous rapamycin treatment as a positive control to assess whether intermittent rapamycin improved outcomes to levels observed under continuous treatment. We used the highest rapamycin dose previously described to extend lifespan in both males and females [42 mg/kg diet) [9] and provided rapamycin in the food every other week. Intermittent rapamycin feeding increased serum rapamycin levels while the mice were on the drug (‘inter-on’), although not to the levels observed in continuously fed animals, while levels quickly fell to those of controls after one week off the drug (‘inter-off’). Concentrations in other tissues and their responses to weeks on and off intermittent treatment were sex- and tissue-specific. The adverse effects of a high dose chronic rapamycin treatment on glucose tolerance were ameliorated in inter-on females and inter-off animals of both sexes and were even restored to control levels in inter-off females. However, the effect of the drug on testicular pathology was not rescued by intermittent treatment. Both treatment regimes resulted in a comparable increase in heart fibrosis in males and liver lipidosis in both sexes, particularly females. Hence, these adverse effects of rapamycin treatment persisted to some extent with intermittent treatment. Intermittent and continuous rapamycin treatments increased lifespan to the same extent in males, while intermittently fed females were slightly shorter lived than continuously treated animals. In males, the two treatment regimes led to a similar increase in motor coordination, maintenance of heart rate and Q-T interval and reduction in spleen weight. In females, reduced BAT inflammation and spleen weight and maintenance of heart rate and Q-T interval were similar under the two regimes. These traits are therefore candidate contributors to the increased lifespan resulting from rapamycin treatment. Other health parameters, including age related pathologies, were either unaffected or influenced to a greater extent by continuous treatment. Finally, the abundance of circulating immune proteins was differentially regulated depending on sex and drug dosing regimen. The significantly regulated proteins were mainly downregulated under continuous treatment, with more changes detected in females than in males. In line with the impact of treatment regimen on tissue inflammation, intermittent rapamycin resulted in fewer significant changes, and in many regulated proteins, the magnitude of the effect was less than under continuous treatment. Hence, intermittent rapamycin treatment was effective in prolonging lifespan and reduced some, but not all, side-effects of chronic treatment, but chronic treatment was more beneficial to healthspan.

## 2 Results

### 2.1 Effects of continuous and intermittent rapamycin dosing on tissue concentrations

**Figure 1:**
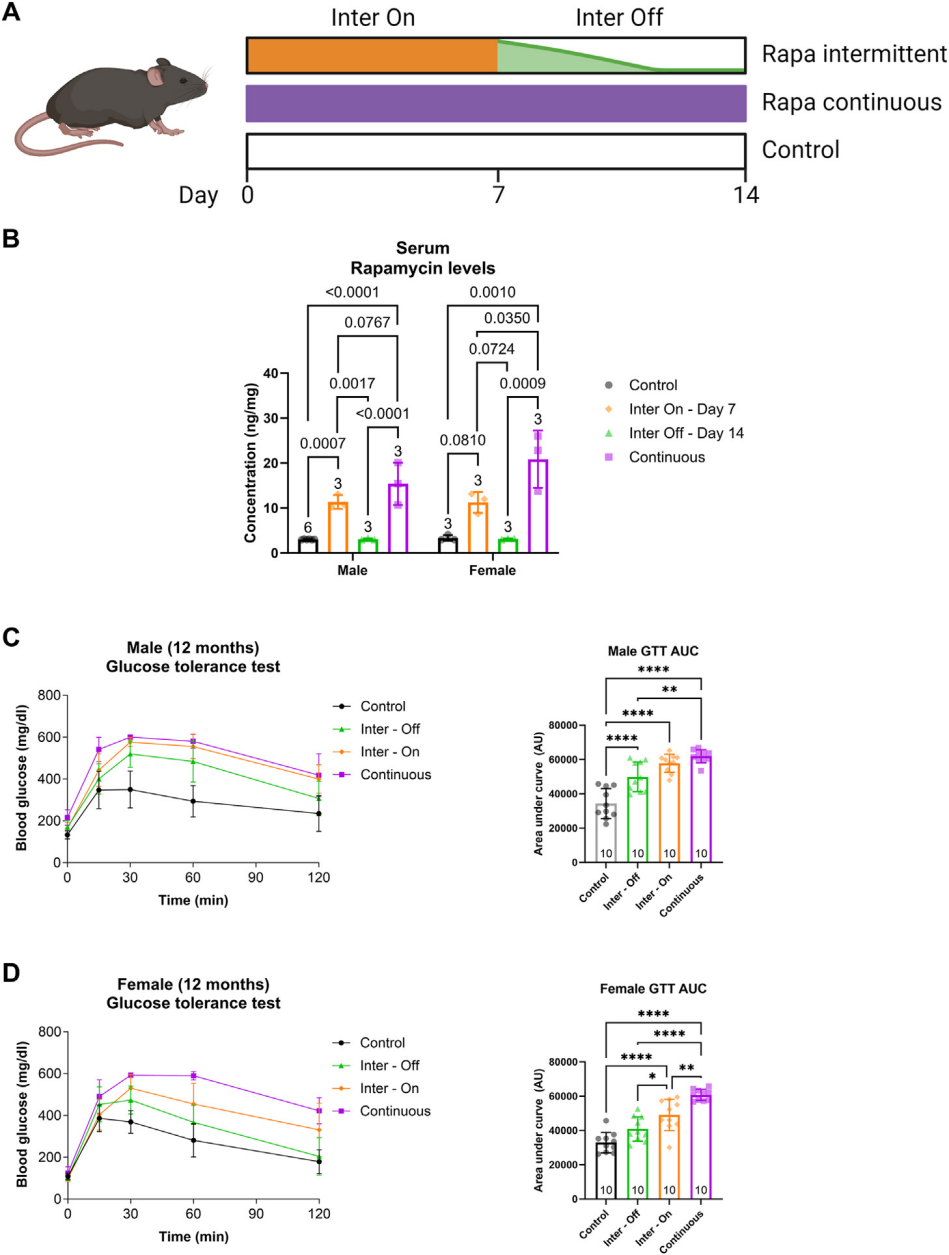
Effects of continuous and intermittent rapamycin dosing on glucose tolerance. (A) Schematic overview of the rapamycin treatment protocol. Inter-Off mice were measured after 7 days on control chow, while Inter-On mice were measured after 7 days on rapamycin food. (B) During the ‘off’ period of intermittently treated mice, there was a significant reduction in rapamycin concentration in serum of both sexes. (C) Blood glucose levels of 12 months old male C3B6F1 mice was plotted against time after glucose injection (left panel). Area under the curve (AUC) analysis of male glucose tolerance test (GTT) revealed significantly lower glucose tolerance of continuously rapamycin fed mice, which was partially rescued with intermittent rapamycin feeding during weeks on control chow, i.e. inter-off, while no improvement was measured when mice were back on rapamycin chow, i.e. inter-on. (D) GTT of 12 months old C3B6F1 female mice. AUC analysis revealed significantly lower glucose tolerance due to continuous rapamycin treatment. This was partially rescued by intermittent rapamycin feeding during inter-on time periods, and glucose sensitivity was further improved to levels comparable to control mice when mice were fed control chow (inter-off). Number of animals reported at the bottom of the bars. Error bars indicate SD. Differences in serum rapamycin levels and glucose tolerance AUC were detected using one-way ANOVA followed by Holms-Sidaks and Tukey test respectively for multiple comparisons. ∗*P* < 0.05, ∗∗*P* < 0.01, ∗∗∗*P* < 0.001, ∗∗∗∗*P* < 0.0001. Detailed statistical values found in Table S1.

We used male and female C3B6F1 hybrid mice [31] in this study to provide a robust, heterozygous background to assess animal physiology without confounds due to a particular inbred line on longevity [32]. Mice fed with control (Eudragit S100 at 0.48 g/kg diet), intermittent (every other week feeding) or continuous rapamycin (42 mg/kg diet) regimens starting from 10 months of age were used to test whether intermittent rapamycin feeding is sufficient to reach the serum concentrations seen in continuously dosed mice and to reduce, or fully wash out, rapamycin levels when the drug was withdrawn (Figure 1A). Rapamycin levels were measured by mass spectrometry in serum and selected tissues of intermittently treated mice while they were on the rapamycin food (inter-on) and after one week of rapamycin withdrawal on the control food (inter-off), and of continuously treated animals and controls.

Inter-on male serum rapamycin levels were significantly higher than controls and inter-off levels, and not significantly different from continuously fed males, indicating that during the on weeks males experienced a drug dose similar to continuously treated males (Figure 1B). Inter-on female rapamycin levels were non-significantly higher than controls and inter-off levels, and the levels were also significantly lower than in continuously fed females (Figure 1B). Thus, while intermittent treatment increased circulating serum levels in females, one week was insufficient to reach similar levels as continuously fed females. In summary, on-week males but not females took up a drug dose similar to continuously treated animals. One week off rapamycin was efficient to deplete circulating rapamycin levels in both sexes to those seen in untreated controls (Figure 1B).

We also assessed drug levels in liver (Figure S1A–B), white adipose tissue (WAT, Figure S1C–D) and brain (Figure S1E–F). Rapamycin levels in liver of both sexes were similar between continuously dosed and inter-on animals, but declined to barely detectable levels after one week of drug withdrawal, indicating efficient wash out of rapamycin from the liver (Figure S1A–B). In the WAT, rapamycin levels were significantly decreased in female (Figure S1D) but not male mice (Figure S1C) by one week of drug withdrawal, and in females the drug concentration of inter-on animals did not reach those seen with continuous treatment. In the brain of both sexes, drug levels in the intermittently dosed animals were similar in inter-on and -off, and considerably lower than in the continuously dosed animals (Figure S1D–E), suggesting that animals have to be dosed for more than a week to achieve an equilibrium concentration. While the overall levels of rapamycin in the brain and WAT were similar, the liver showed an about tenfold higher concentration. Furthermore, we did not detect any significant effect of sex on rapamycin levels in serum (Figure S1G), liver, WAT, or brain. In summary, the intermittent feeding regime was sufficient to raise serum and liver levels of rapamycin to continuous levels during on weeks and reduce drug levels during off weeks, while in male as well as female WAT and brain there were time lags.

### 2.2 Effects of continuous and intermittent rapamycin dosing on metabolic health

Rapamycin can induce multiple beneficial phenotypes. However, not all phenotypes are desirable, including an increased incidence of cataracts [17], testicular degeneration [17], glucose intolerance [9,18] and reduction in blood T regulatory cells [19]. We therefore carried out detailed phenotyping to detect whether any of these were reduced by intermittent administration of the drug. Chronic rapamycin treatment with 42 mg/kg of diet reduces glucose tolerance [9], while intermittent rapamycin treatment with a lower dose (2 mg/kg of diet) does not, while extending lifespan in females [26]. We performed a glucose tolerance test in middle-aged (12-month-old) male and female mice treated continuously or intermittently for 2 months with rapamycin at 42 mg/kg of diet (Figure 1C,D). Continuously dosed animals of both sexes had lower glucose tolerance than controls. Intermittently fed inter-on females, but not males, showed significantly increased glucose tolerance compared with continuously fed animals, with both sexes showing lower tolerance than control animals. Inter-off females, but not males, showed similar glucose tolerance to controls, and both sexes showed increased tolerance relative to continuously fed animals. The adverse effects of a high dose chronic rapamycin treatment on glucose tolerance were thus ameliorated in inter-on females and inter-off animals of both sexes, and were restored to control levels in inter-off females. Fasted blood glucose levels were significantly increased in continuously fed males (Figure S2A), but not in females (Figure S2B). In inter-on animals of both sexes there was no difference from control, with increased blood glucose in inter-off males. In summary, these results indicate that chronic rapamycin feeding negatively affects glucose metabolism, an effect that can be ameliorated and sometimes abolished by an intermittent feeding regime.

In contrast to glucose tolerance, we did not observe significant effects of rapamycin on insulin tolerance in either continuously or intermittently fed C3B6F1 mice at 12 months of age (Figure S2C and D). However, it is important to note that at this age control C3B6F1 male and female mice were already mostly resistant to the injection of insulin, preventing us from determining the detrimental effect of rapamycin on insulin sensitivity.

### 2.3 Effects of continuous and intermittent rapamycin dosing on body weight and lifespan

Continuous rapamycin treatment reduces age-associated body weight gain in both sexes [9]. Consistent with previous findings [9], continuously treated animals were resistant to the age-related weight gain observed in control animals (Figure S3A and B). Intermittently treated male mice showed a small attenuation in body weight gain (Figure S3A), but they were significant heavier than continuously treated males. Intermittently fed females did not differ in weight from control animals (Figure S3B). Thus, intermittent rapamycin feeding did not affect body weight as much as chronic treatment.

**Figure 2:**
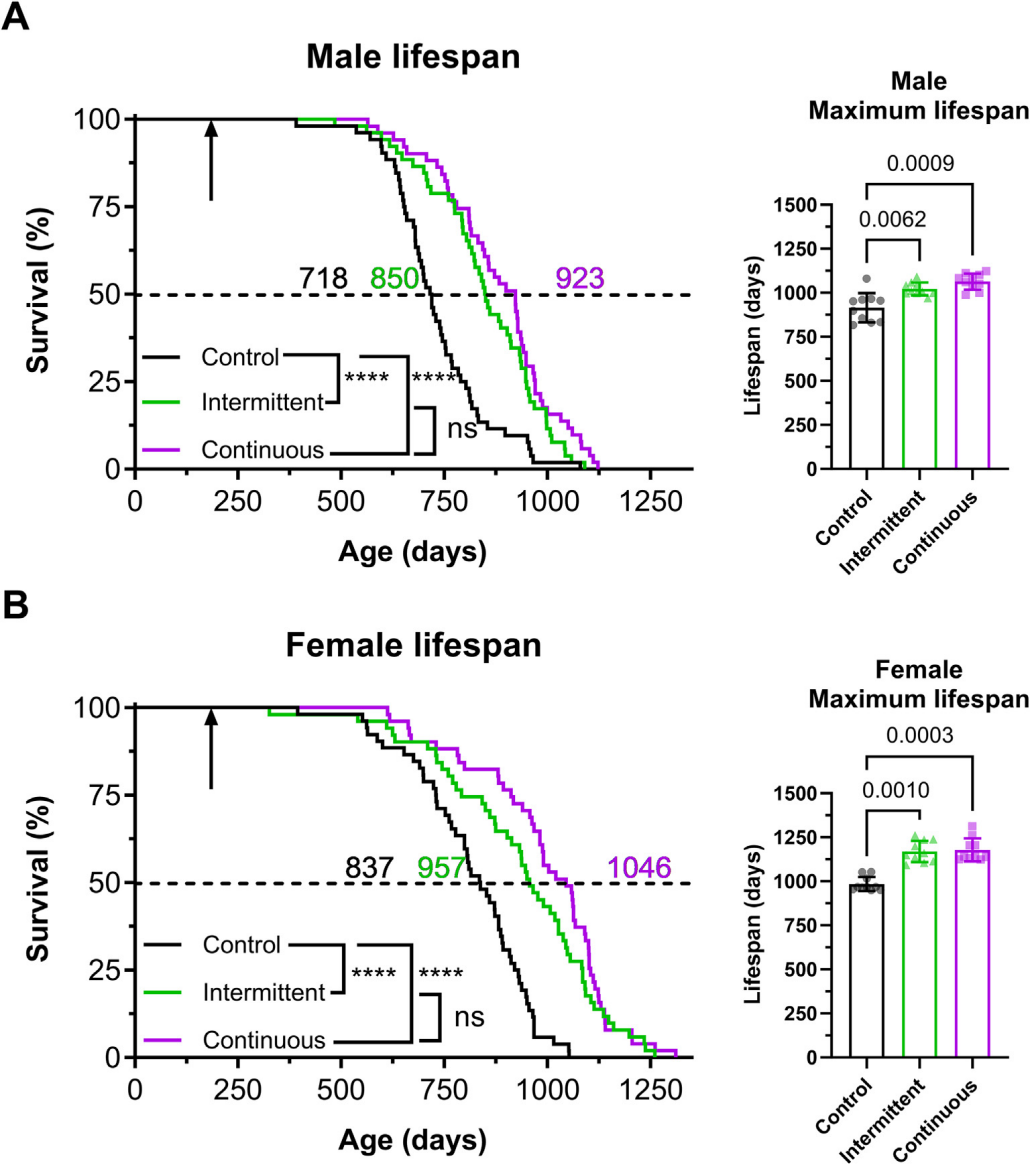
Intermittent administration of rapamycin extends lifespan to a similar extent to continuous treatment in males, and slightly less in females. Kaplan–Meier survival plot depicting survival of (A) male and (B) female C3B6F1 hybrid mice under intermittent and continuous rapamycin feeding. Intermittent rapamycin feeding led to a significant extension in median and maximum lifespan compared to control mice in both males and females. While in males continuously and intermittently treated animals showed a similar lifespan extension, in females continuously treated animals were slightly longer-lived. (Male controls *n* = 52, intermittent *n* = 52 and continuous *n* = 51, female controls *n* = 52, intermittent *n* = 51 and continuous *n* = 51). ∗*P* < 0.05, ∗∗*P* < 0.01, ∗∗∗*P* < 0.001, ∗∗∗∗*P* < 0.0001, Log rank test. Black arrow indicates the start of the rapamycin treatments, coloured numbers note the median lifespan. For detailed statistics see Table S1.

In male C3B6F1 mice, both intermittent and continuous rapamycin feeding regimens led to increases in median lifespan, of 18% and 28% respectively (Figure 2A). Moreover, comparisons using pairwise log-rank test between intermittent and continuously fed mice did not detect any significant difference between treatment groups (*P* = 0.13). However, given the shape of the survival curves and the small effect size observed between intermittent rapamycin and the continuous group in male mice, we would need a total of 373 males in both groups to detect a difference this small. The statistical power to detect a difference of this magnitude was not achieved in our experimental design, as the calculated power for detecting this difference was determined to be 0.314. To determine whether rapamycin treatment extended maximum lifespan we computed the 90th percentile age of all three groups of mice and performed the Wang–Allison test [33]. We found that both intermittent and continuous rapamycin treatment extended maximum lifespan, with no significant difference between the two drug regimens (Figure 2A). In female mice, both intermittent and continuous regimens led to a significant increase in median lifespan, of 14% and 25% respectively (Figure 2B), compared to controls. Survival curves of the continuous and intermittent feeding groups crossed at day 1140, which reduces the chance of detecting a difference using the log-rank test [34]. Therefore, we performed a restricted mean time lost (RMTL) analysis, where a model-free contrast is performed of time (life) lost in two groups at a chosen time. We compared RMTL between intermittent and continuous feeding before the time of crossing or day 1130. Using this approach, we did not detect any significant difference between the RMTL of continuous rapamycin feeding and intermittent feeding (Figure 2B), but there was a trend (*P* = 0.082). Therefore, we added mice from the phenotyping, tissue collection and pathology cohorts in this study and repeated the analysis (Figure S4), and found a significant difference in RMTL between intermittent and continuous (*P* = 0.015) fed females. Similar to males, maximum lifespan was also extended by both rapamycin feeding regimens in females, with no significant difference between regimens (Figure 2B). Further analysis did not detect any significant interaction between treatment and sex (*P* value for Intermittent: Sex = 0.28481 and P value for Continuous: Sex = 0.26505). In summary, intermittent and continuous feeding extended median and maximum lifespan to a similar extent in males, while the continuously fed females were slightly longer-lived than the intermittent, with no significant difference in the degree of extension between the sexes.

### 2.4 Effects of chronic and intermittent rapamycin feeding on motor coordination, running endurance and exploratory activity

**Figure 3:**
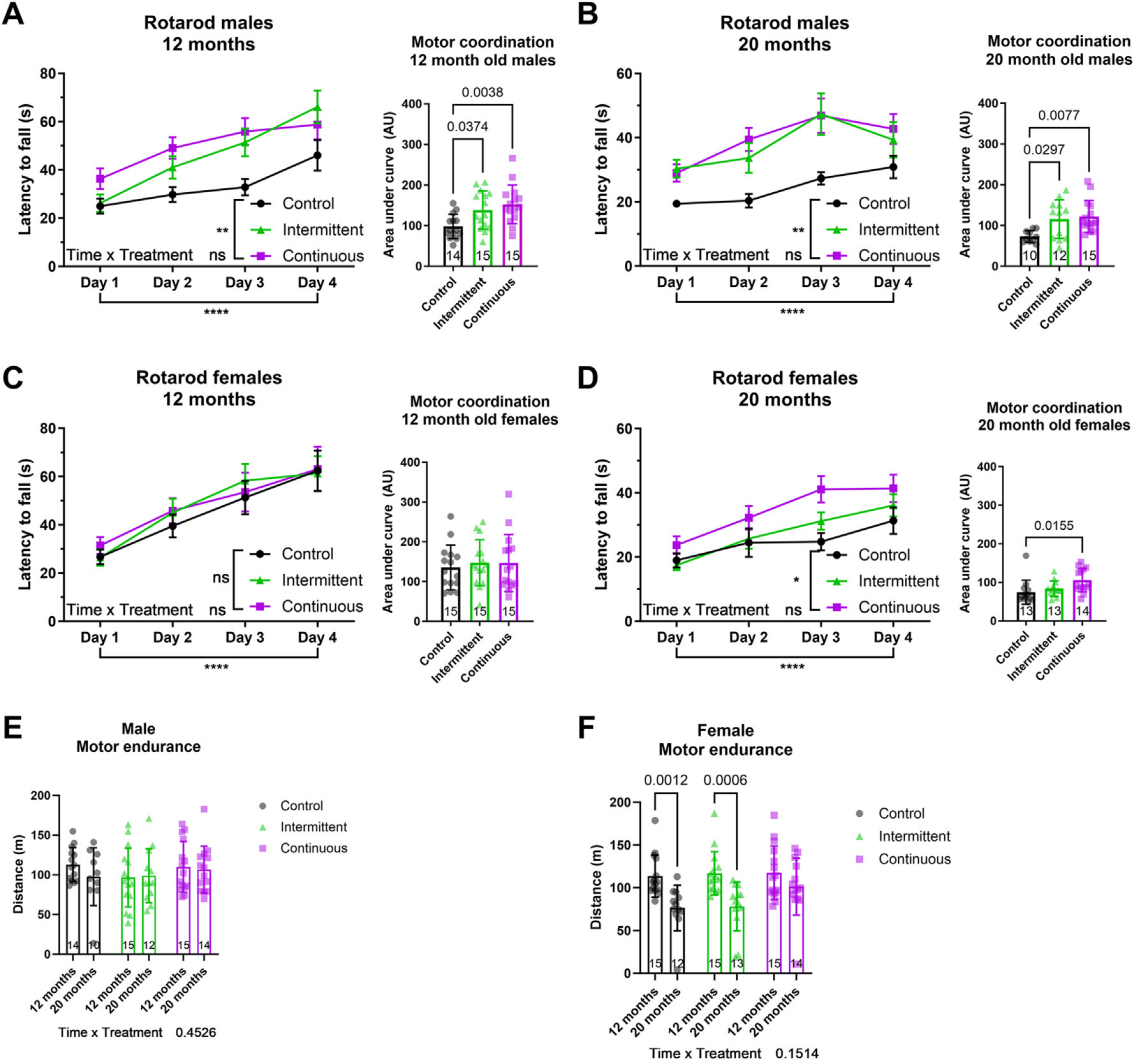
Intermittent and continuous rapamycin feeding improves motor coordination in males independent of age. Average latency to fall in the Rotarod revealed a significant improvement in motor coordination in (A) 12 months and (B) 20 months old male C3B6F1 mice dosed intermittently and continuously with rapamycin. (C) Rapamycin treatment in 12 months old females mice did not significantly change motor coordination. (D) Continuous but not intermittent rapamycin treatment significantly improved motor coordination in old female mice. The maximum distance run by (a) male and (b) female C3B6F1 mice until exhaustion was measured by treadmill. (E) No significant difference in running endurance due to age or treatment were observed in male mice. (F) Age-associated decline in running endurance was observed in female mice, but no effect of rapamycin treatment was detected. Interaction between age and treatment was analysed by mixed-effects model, followed by Sidaks post hoc test. Error bars denote SEM. ∗*P* < 0.05, ∗∗*P* < 0.01 and ∗∗∗∗*P* < 0.0001. Two-way ANOVA. For detailed statistical values see Table S1.

Chronic rapamycin treatment increases physical health of mice at old age [8]. Therefore, we measured motor coordination by rotarod, running endurance by treadmill and exploratory drive by open field assays in 12 months (middle-aged) and 20 months (old) rapamycin-treated mice. Both continuously and intermittently treated male mice remained on the rotating rod significantly longer than controls at middle (Figure 3A) and old age (Figure 3B), with no significant difference between them. In contrast, rotarod performance was not significantly improved by rapamycin feeding in middle-aged females (Figure 3C) and at old age only chronic rapamycin treatment did so (Figure 3D). We next measured forced running endurance using a treadmill assay, but found no changes in male endurance with age or due to rapamycin treatment (Figure 3E). In females, no effect due to rapamycin treatment was detected, although we observed an age-associated decline in endurance (Figure 3F). In the open field assay, there were no significant changes upon rapamycin feeding on the exploratory behaviour of middle-aged mice of both sexes (Figure S5A and B). At old age, chronic rapamycin feeding significantly increased the exploratory drive, measured by distance travelled and speed in the open field arena, of both male and female mice (Figure S5C and D), while intermittent feeding showed an in-between phenotype in male but not female mice. Noteworthy, neither chronic nor intermittent rapamycin feeding affected centre occupancy in the open field arena of male or female mice at middle or old age, suggesting that rapamycin did not affect anxiety-like behaviour. In summary, while intermittent rapamycin feeding was able to improve motor coordination in males, the chronic rapamycin treatment showed more beneficial effects than did intermittent feeding on physical health parameters.

### 2.5 Rapamycin treatment reduced age-related changes in heart function

**Figure 4:**
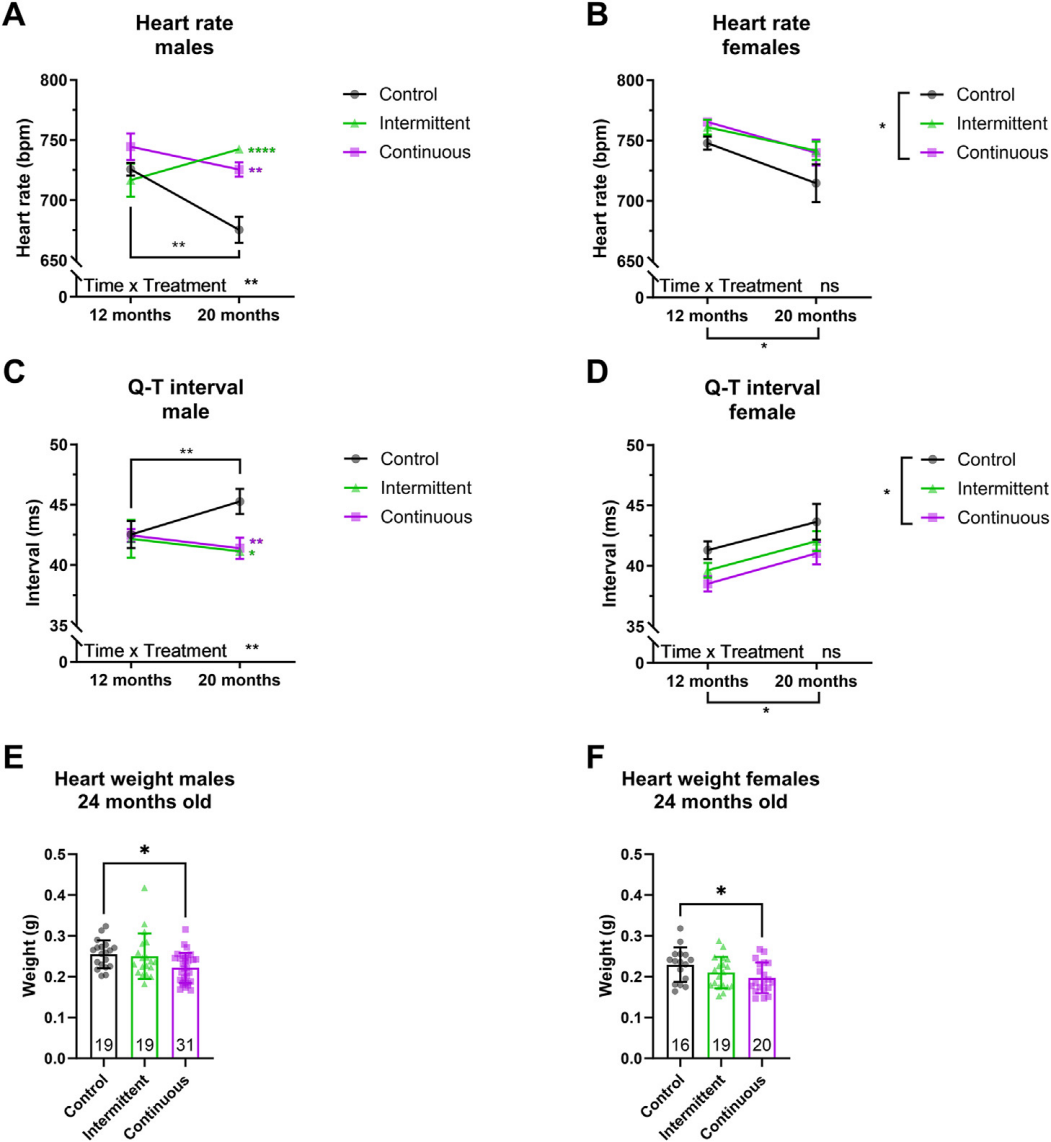
Intermittent and continuous rapamycin treatment improves heart parameters at old age. (A–B) Heart rate and (C–D) length of Q-T interval measured by non-invasive electrocardiography in (A, C) male and (B, D) female C3B6F1 mice fed continuously or intermittently with rapamycin. Heart rate compared to control animals was increased by rapamycin treatment at old age, while Q-T- interval length was decreased. (E–F) Post mortem heart weight of 24 months old (E) male and (F) female mice. Heart weight was significantly reduced by chronic rapamycin feeding in males and females. ECG: *n* = 8 male mice per treatment group and age, female control middle-age *n* = 15 and old *n* = 9, female intermittent regimen middle-age *n* = 15 and old *n* = 10, and female continuous treatment middle-age n = 14 and old n = 8. Error bars indicate SEM for a–d and SD for E–F. Differences in longitudinal data were tested with a mixed-effects model followed by Tukey test for multiple comparisons. One-way ANOVA was used to test differences in heart weight, followed by Tukey post hoc test.∗*P* < 0.05, ∗∗*P* < 0.01, ∗∗∗*P* < 0.001, ∗∗∗∗*P* < 0.0001. For detailed statistical values see Table S1.

Mice display cardiac changes similar to those observed in aging humans [35]. To address the impact of intermittent rapamycin feeding on age-related heart function, we measured heart rate in middle-aged and old mice using non-invasive electrocardiography (ECG). In control animals, heart rate decreased between 12 and 20 months of age, both in males (Figure 4A) and females (Figure 4B). Animals treated continuously or intermittently with rapamycin showed a reduction in age-associated heart rate decrease at 20 months of age, with no significant differences between them (Figure 4A,B). To pursue this further, we looked at the specific parameters of the ECG, especially the QT interval, which is an outcome of increased left ventricular hypertrophy (LVH), a major cause of cardiovascular disease and early death worldwide [36]. Previous studies have reported that short-term rapamycin treatment in mice can reduce the age-related increase in LVH [37,38]. Consistent with these findings, continuous and intermittent rapamycin treatment both completely rescued the age-associated increase in QT interval length in males (Figure 4C) and reduced it in females (Figure 4D). Interestingly, rapamycin-treated female mice already showed a significant reduction in QT interval at 12 months of age (Figure 4D), suggesting that rapamycin treatment in females reduces QT interval in an age-independent manner, while males only showed differences at old age (Figure 4C). Finally, we determined heart weight of mice under intermittent and continuous rapamycin feeding at 24 months of age. We observed a significant reduction in heart wet weight in continuously but not intermittently fed males (Figure 4E) and females (Figure 4F). In summary, these results suggest that both chronic and intermittent rapamycin feeding significantly, and to a similar extent, slow the advancement of age-related cardiac outcomes such as heart rate and LVH.

### 2.6 Rapamycin treatment did not significantly affect energy metabolism

There were no significant changes in energy expenditure measured via indirect calorimetry upon chronic or intermittent rapamycin treatment during daytime or nighttime in 16-month-old mice (Figure S6A–D). Indirect calorimetry also detected no effect of rapamycin treatment on the respiratory exchange ratio (RER) in males (Figure S6E), while in females only continuously but not intermittent rapamycin feeding increased the RER during the day, with a similar but non-significant trend at night (Figure S6F). An increase in RER suggests female mice fed continuously with rapamycin have shifted metabolism towards carbohydrate utilisation and away from lipid oxidation. An increase in RER due to rapamycin treatment has been observed previously in aged female [26] and male [39] mice.

### 2.7 Chronic but not intermittent rapamycin treatment reduces age-associated pathology

To address whether chronic and intermittent rapamycin treatment affect age-associated pathologies in a similar manner, we performed a detailed histological assessment of heart, liver, kidney, brown adipose tissue (BAT), pancreas, white adipose tissue (WAT) and spleen tissue collected from mice at 24 months of age.

**Figure 5:**
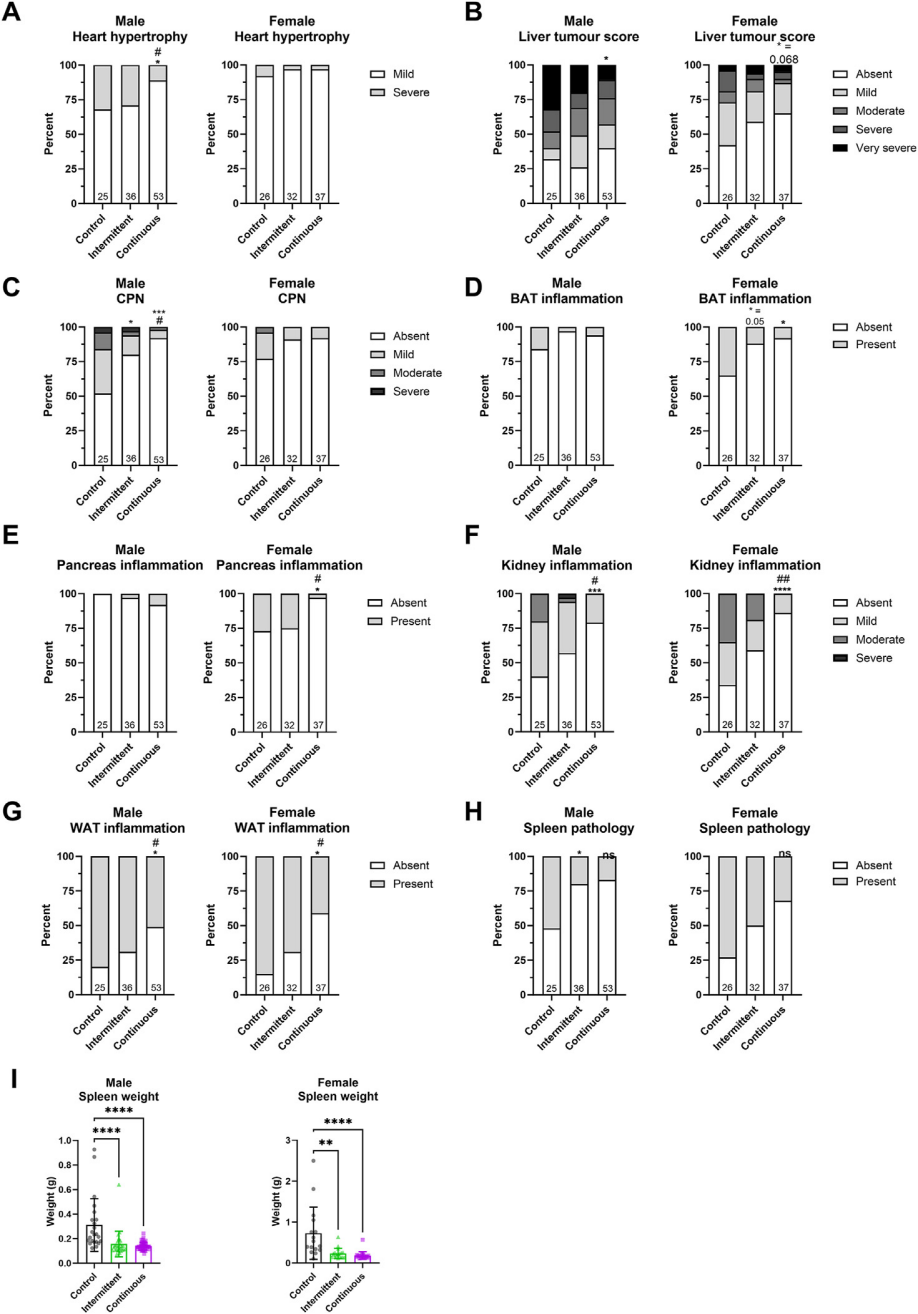
Rapamycin treatment reduced organ pathology in late life. Histopathological analysis of 24 months old male and female C3B6F1. (A) A significant reduction in heart hypertrophy was observed in continuously but not intermittently rapamycin fed mice. (B) Liver tumour score was significantly reduced in continuously fed male mice compared to controls. (C) Chronic progressive nephropathy (CPN) was significantly reduced in males intermittently fed with rapamycin, and further reduced upon continuous feeding. (D) Inflammation levels in brown adipose tissue (BAT) were reduced in males and females in response to intermittent and continuous rapamycin feeding. (E) Pancreas inflammation in females was ameliorated by continuous but not intermittent rapamycin feeding. (F) Kidney and (G) white adipose tissue (WAT) inflammation was significantly reduced upon continuous rapamycin treatment in male and female mice, while the intermittent treatment only showed a trend towards reduced inflammation. (H) Chronic and intermittent rapamycin feeding significantly reduced spleen pathology in males and a similar trend was observed also in females. (I) Spleen weight was significantly reduced in males and females treated with rapamycin continuously or intermittently. Asterisks denote the following relative to control: ∗*P* < 0.05, ∗∗*P* < 0.01, ∗∗∗*P* < 0.001, ∗∗∗∗*P* < 0.0001. Hashtag denote the following relative to intermittent rapamycin: #*P* < 0.05 and ##*P* < 0.01. For detailed statistical values see Table S1.

We observed reduced heart hypertrophy in continuously, but not intermittently, fed old males (Figure 5A). However, we did observe a significant increase in heart fibrosis in both intermittent and continuously fed males (Figure S7A). Interestingly, we found no evidence of heart hypertrophy (Figure 5A) or heart fibrosis (Figure S7A) in old female controls at 24 months of age. A previous study did not find evidence of altered collagen ratio, also suggesting no change in fibrosis in response to rapamycin treatment in old female mice [37].

In the liver, we detected a significant reduction in the percent of mice with severe tumours in continuously treated males, and a similar trend in females (*P* = 0.068) (Figure 5B), consistent with the hypothesis that continuous rapamycin treatment delays neoplastic liver disease [10,39]. Intermittent rapamycin treatment did not significantly reduce tumour incidence in males or females compared to controls but presented as an in-between phenotype (Figure 5B), suggesting that continuous rapamycin treatment is more efficient in suppressing liver tumorigenesis. Liver lipidosis was increased in both intermittent and continuously fed males and females (Figure S7B), consistent with previous reports [40].

Chronic progressive nephropathy (CPN) is an age-associated disease prevalent in rodents that affects glomerular filtration rate with potential adverse effects on animal health [41]. Both intermittent and continuously rapamycin fed males had significantly reduced levels of CPN compared to controls, with a larger effect of continuous rapamycin (Figure 5C). CPN was also reduced in female mice treated with rapamycin, however, given the low CPN levels in control animals this effect was not significant and there was no difference between intermittent and continuous rapamycin feeding (Figure 5C). In contrast to CPN, rapamycin had no effect on kidney glomerulopathy in males or females (Figure S7C).

Another adverse effect of rapamycin treatment is an increase in male gonadal pathology [39], reversible on withdrawal of the drug [42]. Intermittent rapamycin treatment caused similar gonadal pathology as continuous rapamycin feeding (Figure S7D), indicating that the intermittent feeding regime is not protective in this context.

Inflammation is characterized by the accumulation of white blood cells within tissues. Continuous rapamycin feeding significantly reduced inflammation in BAT, pancreas, kidney and WAT of female mice (Figure 5D–G). In contrast, intermittent rapamycin feeding was not as effective in reducing inflammation in female mice, having no effect on pancreas inflammation and showing only a non-significant trend in reducing BAT, kidney or WAT inflammation. Apart from the WAT, tissue inflammation in males was not as pronounced as in females. Nevertheless, continues rapamycin feeding significantly reduced inflammation in the male kidney and WAT and showed a trend in the BAT. As in females, intermittent rapamycin treatment was less effective in reducing inflammation and only showed a non-significant trend towards reduced inflammation in WAT, kidney and BAT. Thus, continuous rapamycin treatment effectively reduced tissue inflammation in males and females, while the intermittent feeding regime was less effective.

The spleen is one of the primary sites of immune cell production [43]. Interestingly, we found a significant reduction in spleen pathology, as measured by cellular composition of the different splenic regions [44], in both intermittently and continuously rapamycin fed males (Figure 5H). Continuously fed females also presented with a significant reduction in spleen pathology (Figure 5H), while intermittent fed females showed a trend (*P* = 0.078). Splenomegaly, or enlargement of the spleen, is a phenotype that can be caused by multiple conditions such as hematologic cancers, as neoplastic cells infiltrate the spleen, and is typically seen in advanced age [45]. We measured spleen weight, and found a significant reduction in males and females under both continuous and intermittent rapamycin feeding indicating a reduction in splenomegaly symptoms in these animals (Figure 5I). Intermittent and continuous rapamycin feeding thus significantly reduce spleen pathology in males and weight in both sexes to the same extent, while in females, spleen pathology was not as strongly improved as with the continuous treatment.

Overall, these data suggest that intermittent rapamycin treatment can reduce late-life pathology, but in general does so less than in continuously fed mice.

### 2.8 Rapamycin feeding reduced the levels of circulating immune-related proteins in old mice

**Figure 6:**
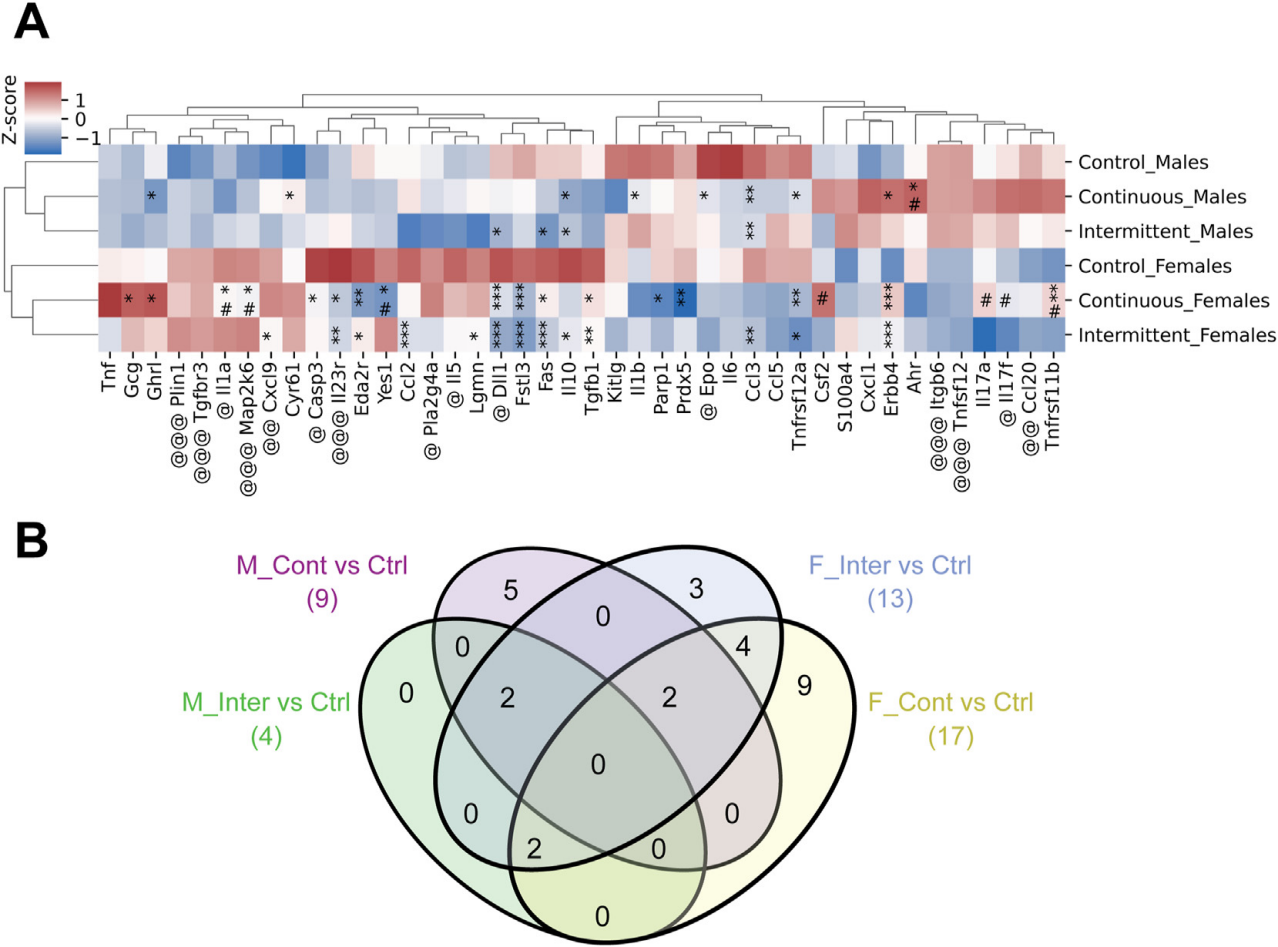
Rapamycin treatment regulates plasma immune process proteins. Circulating proteins were measured using the Olink platform in plasma samples of 24-month-old male and female C3B6F1 mice. (A) Hierarchical clustering of immune process genes leads to separation by sex then by treatment of Olink results. (B) Venn diagram of differentially abundant immune system plasma proteins upon rapamycin feeding in male and female mice. Comparing plasma from intermittent vs control (Inter vs Ctrl), continuous vs control (Cont vs Ctrl) in male and female mice. Total number of differentially abundant, treatment-specific proteins are shown in parentheses. ∗ significant compared to control, # significant compared to continuous, @ significant between male and female controls. *P* values: ∗ <0.05, ∗∗ <0.01, ∗∗∗ <0.001. For detailed statistical values see Star Methods.

Continuous rapamycin feeding reduced age-associated inflammation in multiple tissues, whereas intermittent rapamycin feeding was less effective in doing so (Table 2). We therefore measured levels of circulating immune-related proteins using the mouse explanatory panel from Olink® [46] in the plasma of 24-month-old male and female mice upon chronic and intermittent rapamycin feeding (Figure S8A and B). Euclidean clustering based on the 92 measured proteins, grouped animals according to sex and response to rapamycin treatment. Furthermore, animals fed with rapamycin continuously and intermittently grouped together, indicating that their plasma proteomes were more similar than to the controls (Figure S8A and B). We next focused on the 42 immune-related proteins, where we observed a similar sex- and treatment-specific separation pattern (Figure 6A). Interestingly, in females (17/13, continuous/intermittent) more immune-related proteins were significantly regulated in response to rapamycin treatment than in males (9/4, continuous/intermittent), with the majority down-regulated in response to rapamycin. This may indicate that rapamycin held back the age-related increase of these immune-related proteins, because some of them are upregulated with age in mice, such as Ccl5, Ccl20, Eda2r, Ghrl, Il1b, Il5, Lgmn and Tnf [47]. Plasma biomarkers of ageing are highly enriched in SASP factors [48] and rapamycin treatment reduced SASP both in vitro [49] and in vivo [50], suggesting that the observed changes in the plasma proteome might indicate changes in the SASP. Of the 42 immune-related proteins measured here, 13 proteins were associated with the SASP including Tnf, Il1α, Il1β, Il6, Ccl2, Ccl3, Ccl5, Ccl20, Cxcl1, Cxcl9, Map2k6, Cyr61 and Tgf1β. Among these, Ccl3 was down-regulated in intermittent and continuously treated males and females, Ccl2 and Tgfb1 were down-regulated in intermittent and continuously treated females and Il1a and Map2k6 were only significantly regulated in continuously treated females. Thus, rapamycin treatment reduced plasma levels of SASP associated proteins in a sex and treatment specific manner, whereby effects were stronger in female mice and the continuous treatment was more effective than the intermittent regime in reducing SASP protein levels. Changes in immune-related proteins were not restricted to the SASP, suggesting that rapamycin affects the plasma proteome via different mechanisms. While most proteins were present at lower level upon rapamycin treatment, plasma levels of receptor tyrosine-protein kinase Erbb4 was more abundant in male and female mice treated continuously and intermittently with rapamycin. Increased levels of ERBB4 in mesenchymal stem cells have been shown to have beneficial effects on stem cell rejuvenation and heart function in old mice [51], implicating it as a potential systemic downstream effector of rapamycin. There was only little overlap in significantly regulated proteins between males and females in the response to rapamycin feeding (Figure 6B), with only Tnfrsf12a and Erbb4 shared between continuously-treated and Ccl3, Fas, Il10 and Dll1 shared between intermittently-treated males and females, indicating that the effect of rapamycin on the plasma protein is very sex-specific. In summary, rapamycin treatment reduced the levels of circulating immune-related proteins including SASP components, more in females, and with more pronounced effects in continuously treated animals compared to the intermittent feeding regime.

## 3 Discussion

We have assessed whether intermittent dosing with rapamycin can avoid some or all of the negative effects of chronic dosing while recapturing some or all of the beneficial effects. We dosed rapamycin at 42 mg/kg in the food, which has so far resulted in the highest extension of lifespan in both male and female mice [9]. Dosing started at six months of age, with intermittent dosing every other week and we used the robust C3B6F1 hybrid strain of mice to avoid the effects of genome-wide homozygosity of inbred strains. We investigated the responses of both male and female mice, and we included the continuously fed, positive control to enable us to assess if the effects of continuous dosing were fully recaptured by intermittent treatment.

We assessed rapamycin concentrations in serum, liver, WAT and brain. Noteworthy, we did not observe sex differences in overall rapamycin serum levels in our C3B6F1 hybrid mice and consistently the effect of rapamycin on lifespan was similar between male and female mice, in contrast to what has been previously reported for UM-HET3 animals [9], suggesting that this sex-specific effect of rapamycin treatment is dependent on the genetic background. Tissue concentrations of rapamycin were both sex- and tissue-specific. Intermittent rapamycin feeding significantly increased tissue rapamycin levels in inter-on compared to inter-off except in the brain, and only in the liver and WAT of males did concentration reach the levels seen in continuously dosed mice. The concentration of rapamycin in the brain was much lower in males than in females, and was lower in both inter-on and inter-off than in continuously fed animals of both sexes. While we did not measure any parameters specific to age-associated cognitive decline, the lack of rapamycin levels in the brain could suggest that intermittent rapamycin feeding may not be sufficient to recapitulate previous reports of benefits to cognitive function [52]. The two treatments therefore did not differ solely in the timing of dosing, but also in the concentrations of drug achieved in two sexes and different tissues. It is possible that longer periods on and off the drugs in the intermittent animals could have achieved similar drug concentrations in tissues, with consequently altered phenotypic effects. Three months of rapamycin treatment can result in improved gut health in mice at least 6 months after the drug has been withdrawn [29], and longer periods of drug treatment and withdrawal may benefit other tissues.

In this study, we used every other week feeding to apply rapamycin intermittently, which differs from others studies in which rapamycin was applied by intraperitoneal injection once per week [19] or three times every other week (Leontieva et al., 2014). The half-life of rapamycin in plasma of male mice has been reported to be approximately 15 h [19], suggesting that trough levels of rapamycin are achieved after three days of drug withdrawal. Thus, in the feeding regime used in this study, mice were exposed to rapamycin for around 10 days followed by 4 days of no or very low drug exposure. Given that we observed some sex-specific effects of the intermittent rapamycin treatment, it would be interesting in future studies to directly compare rapamycin wash out kinetics between male and female mice. In some cases, rapamycin is already prescribed off-label as a preventative therapy to maintain health span in humans, however, dosing and dosing interval varies widely with most people taking rapamycin once per week [53]. The plasma half-life of rapamycin differs between rodents and humans, with a reported mean terminal half-life of rapamycin in humans of 79 h [54]. While we show here that intermittent rapamycin increased lifespan to a similar extent than continuous treatment in mice, the differences in species-specific rapamycin turnover rates should be considered when translating findings from rodents to humans.

Impaired glucose tolerance is a common side-effect of chronic rapamycin treatment [18,55,56], and was also observed in the C3B6 F1 hybrid mice used in this study. Rapamycin negatively affects glucose tolerance by inhibiting insulin production and secretion from pancreatic beta-cells [56,57] and by causing hepatic insulin resistance [18]. This effect is mediated by mTORC2 and is uncoupled from longevity [18]. While mTORC1 displays acute and universal sensitivity to rapamycin, mTORC2 exhibits a comparatively lower sensitivity, necessitating prolonged and chronic exposure to the drug for effective disruption in vivo or in vitro [18,27]. Consistently, intermittent rapamycin treatment by intraperitoneal injection of rapamycin once every week, did inhibit mTORC1 but not mTORC2 activity and improved glucose tolerance and insulin secretion in male mice compared to chronic rapamycin control animals [55]. We also observed a partial rescue of glucose tolerance upon biweekly rapamycin feeding, with stronger effects in females, whereby in males only inter-off animals showed a significant improvement compared to continuously treated animals. The difference in glucose tolerance between inter-on and inter-off animals indicate that the intermittently treated animals regularly switch from a glucose sensitive to a glucose intolerant state. The exact reason for the improved glucose tolerance and the sex-specific effects of the intermittent rapamycin treatment is currently unknown, and should be addressed in future studies by measuring insulin secretion and mTORC2 activity in these animals.

We did not detect a significant difference in insulin sensitivity upon rapamycin feeding at 12 months of age, but the insulin insensitivity observed in control C3B6 F1 hybrids at this age prevented us from determining the detrimental effect of rapamycin on insulin sensitivity. In contrast, rapamycin treatment in C57BL/6 mice resulted in insulin resistance [18], insulin sensitivity was not significantly affected by 4 weeks or 3 months of rapamycin treatment in 6- or 21-month-old HET3 mice [58].

In males, the two treatment regimes led to a similar increase in motor co-ordination, maintenance of heart rate and Q-T interval and reduction in spleen weight, while in females, reduced BAT inflammation and spleen weight and maintenance of heart rate and Q-T interval were similar under the two regimes. Given the complete extension of lifespan in males and the almost complete extension in females, this indicates that these traits are candidate contributors to the increased lifespan resulting from rapamycin treatment.

Many studies have shown that rapamycin treatment increases motor coordination [8,59,60], while others did not detect differences [26,61,62]. We found that continuous rapamycin led to improved motor coordination at middle-age in males and at old age in males and females., but intermittent rapamycin treatment was sufficient to recapitulate the increase in motor coordination only in males. Under laboratory culture conditions, motor co-ordination itself probably plays little role in determining lifespan, although it may have acted as a marker of other health improvements as others have suggested [63].

Heart dysfunction is associated with old age and can be reversed by short-term rapamycin treatment [[37], [38]]. Moreover, short-term rapamycin treatment followed by removal of the drug for 8 weeks showed persistent benefits in diastolic function and reduction in cardiac hypertrophy [64]. We observed similar benefits to age-associated parameters of cardiac function. Although we did not observe clear signs of cardiac hypertrophy in 24-month-old C3B6F1 control females, male controls presented with cardiac hypertrophy at 24 months which was rescued by continuous rapamycin feeding. Interestingly, heart fibrosis was not observed in old control and rapamycin treated females [37], consistent with our study. However, male mice under intermittent and continuous feeding presented with a higher rate of heart fibrosis at 24 months of age in our study. The discrepancy between the increase in male fibrosis in our study and the lack of difference in a previous study [37] could be due to previously described sex and strain differences in heart ageing [65].

We did not detect a significant difference in energy metabolism upon rapamycin feeding when animals were measured at 16 months of age. In contrast, rapamycin treatment for 20 weeks in genetic heterogenous male mice resulted in reduced oxygen consumption as assessed by indirect calorimetry when measured at 8 months of age [66]. Another study using C57BL/6J mice found no significant difference at 20 months of age in males or females, but a difference only materialised later on in very old mice (>24 months) [61]. The discrepancies between the studies might be due to differences in mouse backgrounds and/or the age at which mice were measured. Notably, fitness phenotyping experiments were conducted both during rapamycin and control chow feeding in intermittently fed mice. However, each experiment was exclusively conducted either during the treatment or after the treatment, with no mixing within a single experiment. It would be interesting to compare mice currently on- and off-rapamycin chow in the future, especially in phenotypes were we saw benefits of intermittent rapamycin treatment to identify whether ongoing feeding is necessary to observe the benefits or if the benefits persist off-rapamycin.

Circulating plasma proteins are extrinsic signals secreted by tissues with the potential to regulate organismal ageing and presents a novel avenue of therapeutic intervention [67]. The ageing spleen is a major organ for driving non-cell autonomous inflammation in several systemic tissues [68]. Moreover, treatment of mice with rapamycin improved immune function in mice [68,69] and the elderly [14,70]. The lowered spleen pathology, size and reduced adipose tissue inflammation we observed in response to rapamycin, are potential indicators of reduced age-related immune system impairment. However, the consequences of alternative treatment regimes on rapamycin need to be further explored to recapitulate the anti-aging effects of continuous treatment. To that end, future work should investigate the age-related changes in mice of the circulating immune-related proteins we identified in this study to understand the temporal dynamics of these proteins. Moreover, the sex differences in immunity need to be considered when designing successful treatment modalities for patients in the future [71].

Other health parameters, including age related pathology, were either unaffected or influenced to a greater extent by continuous treatment. Given the role of rapamycin as a tumour suppressor drug in the clinic and in mice [13,62,72,73], it is likely that rapamycin-mediated increases in lifespan reflect antitumour effects given the majority of lab strains die with some neoplastic disease [74]. The cross-sectional histopathology approach helped us to determine if the lifespan improvement was due to a global inhibition of neoplastic disease or an anti-aging effect with multiple benefits. However, a previous report could not find evidence that rapamycin led to a dramatic change of proportion of different neoplastic disease in continuously treated animals [8]. We saw consistent effects of continuous rapamycin feeding on reduced tumour incidence as reported in previous studies [[8], [17]]. A previous study on intermittent rapamycin treatment in young (2 month old) female 129/Sv mice showed significantly reduced tumour growth [13], while we did not observe these benefits but only a trend, it could be due to the relatively later starting point of rapamycin feeding in our study (6 month old mice). Interestingly, even though the majority of benefits observed were mediated by continuous rapamycin treatment, there were trends of BAT, kidney and WAT inflammation in intermittent fed mice that we may have been underpowered to detect. The difference in tissue pathology of females could be contributing to the incomplete overlap of survival curves between intermittent and continuous rapamycin feeding. Moreover, these data may suggest that the pathology phenotypes in males are not necessary for intermittent feeding to recapitulate continuous feeding lifespan extension.

Inflammation occurs after disruption of tissue homeostasis by cell stress, injury or infection leading to the recruitment of immune cells to resolve the stress. Aging is associated with chronic inflammation [75], which is characterised by cytokine production and is a contributing factor to various age-related diseases [76,77]. mTOR activity is required for pro-tumorigenic senescence-associated secretory phenotype (SASP) production and rapamycin treatment has been shown to reduce expression of SASP cytokines both in vitro and in vivo [49,50,78]. Consistently, several SASP associated proteins were found to be downregulated upon rapamycin treatment in this study, including Ccl3, Ccl2, Tgfb1, Il1α and Map2k6. While we didn't analyse young animals, plasma levels of SASP-associated proteins have been shown to increase with age including Il1α and Ccl3 [48,78], suggesting that rapamycin treatment counteracts the age-related increase in SASP. Noteworthy, the effect of rapamycin on the SASP was very sex-specific, with only Ccl3 being downregulated in both male and female mice. This sex-specific effect in downregulated proteins upon rapamycin treatment might in part be due to differences in the abundance of the regulated proteins in the corresponding control group, possibly caused by male- or female-specific increases of the circulating proteins with age. Consistent with this hypothesis, levels of proteins that were significantly downregulated upon rapamycin treatment in one sex were usually higher in the control animals of that sex compared to the control animals of the other sex. These data emphasize the need to include both sexes when measuring SASP in old organisms. Intermittent rapamycin treatment caused fewer significant changes and for many regulated proteins the effect size was smaller compared to the continuous treatment, consistent with the effect on tissue inflammation. The potential link between circulating proteins and organ pathology should be investigated directly in the future.

Ultimately, the goal of ageing research is to identify treatments that are applicable to humans. mTOR inhibitors are a promising treatment currently undergoing various clinical trials [15].This study reinforces and expands upon previous work that highlight the benefits of reducing the frequency of rapamycin dose to minimise potential detrimental effects [[19], [26]]. However, we observed that while some of the side effects of continuous rapamycin are avoided under the intermittent regimen, so too are some of the health benefits reduced. The underlying molecular mechanisms contributing to these trade-offs need to be directly examined.

## 4 Methods

Key resources table.

**Table:** 

Reagent or resource	Source	Identifier
Chemicals, peptides, and recombinant proteins		
Rapamycin (42 mg/kg diet) encapsulated with Eudragit S100	ssniff Spezialdiäten GmbH	S9159-P776
Eudragit S100 (0.48 g/kg diet)	ssniff Spezialdiäten GmbH	S9159-S712
Insulin (0.75 U/kg of body weight)	Sanofi	01474384
Glucose (10 ml/kg body weight)	DeltaSelect	07462873
Critical commercial assays		
Target 96 Mouse Exploratory panel	Olink Proteomics	95380
Deposited data		
Survival data	This manuscript	Supplementary Table S2
Olink panel results and detailed statistics of male and female C3B6F1 mice	This manuscript	https://github.com/carolinamonzo/MB_proteomics
Experimental models: Organisms/strains		
Mouse: F1 hybrid wild type mice (C3B6F1)	Charles River Laboratories (strain codes 626 and 027)	https://www.criver.com/products-services/find-model/jax-c3hheouj-mice?region=23 https://www.criver.com/products-services/find-model/c57bl6-mouse?region=23
Software and algorithms		
Statistical analysis	stats v4.2.2	R core team [79]
Sample clustering (PCA & PLS-DA)	mixOmics v6.22.0	Rohart et al. [80]
Euclidean clustering and heatmaps	Seaborn v0.12.1	Michael L. Waskom [81]
Gene set enrichment and overrepresentation analysis	Clusterprofiler 4.0	Wu et al. [82]
Survival analysis	Survival v3.5-7	Terry M. Therneau and Patricia M. Grambsch [83]
RMTL analysis	survRM2 v1.0-4	Uno et al. [84]

## 5 Resource availability

### 5.1 Lead contact

Further information and requests for resources and reagents should be directed to and will be fulfilled by the lead contact, Linda Partridge – l.partridge@ucl.ac.uk.

### 5.2 Materials availability

This study did not generate new unique reagents.

### 5.3 Data and code availability

• Raw results of Olink panel have been provided in Supplementary Table S2 and are publicly available as of the date of publication.
• All original code has been deposited on github (https://github.com/) and is publicly available as of the date of publication. Code for data processing and analysis of the Olink panel has been deposited at (https://github.com/carolinamonzo/MB_proteomics).
• Any additional information required to reanalyse the data reported in this paper is available from the lead contact upon request.

## 6 Experimental model and study participant details

### 6.1 Mouse breeding and experimental groups

C3B6F1 hybrids mice [85] were bred in-house by a cross between C3H/HeN females and C57Bl/6N males, which were obtained from Charles River Laboratories. The experimental mice were bred in three cohorts from the same breeding pairs (see Table 1 for details of numbers per cohort and experimental treatment). Directly after birth, larger litters were reduced to a maximum of 8 pups, while litters with fewer than 4 pups were excluded, to reduce any effects of variation in litter size during development. Pups were weaned at 3 weeks of age. Females were individually randomised to cages upon weaning, while males were weaned litter wise, to minimise aggression and fighting. If males of different litters had to be combined, a 2:3 ratio was preferred over a 4:1. All mice were housed in individually ventilated cages, in groups of five mice per cage, under specific-pathogen-free conditions, at 21 °C, with 50–60% humidity and 12 h light/dark cycle. Until 6 months of age, all mice had *ad libitum* access to chow (Ssniff Spezialdiäten GmbH; R/MH, low phytoestrogen, 9% fat, 34% protein, 57% carbohydrates) and drinking water, unless otherwise indicated. At 6 months of age, each breeding cohort was subdivided into three groups. Control animals received chow containing only the encapsulation material, Eudragit S100 (0.48 g/kg diet). In the continuous rapamycin treatment group, animals were fed a diet containing 42 mg of encapsulated rapamycin per kg of diet, a dose that induces significant lifespan extension in both male and female mice [9]. Intermittent rapamycin feeding was implemented by alternating weekly feeding of 42 mg/kg rapamycin food with weekly control feeding.

**Table 1:** Detailed numbers of mice per experimental group as well as time of testing and treatment start. Controls were fed with normal chow containing Eudragit S100 encapsulation material (0.48 g/kg diet). Mice on the intermittent feeding schedule were fed Rapamycin (42 mg/kg diet) one week then one week of normal chow containing Eudragit S100 encapsulation material (0.48 g/kg diet). The continuous feeding group was fed Rapamycin (42 mg/kg diet) continuously throughout the experiment.

Experimental groups	Treatment regimen	Treatment start	Sex	Number of mice
Metabolic phenotyping performed at 11–12 months	Control	10 months	Male	10
			Female	10
	Intermittent		Male	20
			Female	20
	Continuous		Male	10
			Female	10
Fitness phenotyping performed at 12 months and again at 20 months	Control	6 months	Male	15
			Female	15
	Intermittent		Male	15
			Female	15
	Continuous		Male	15
			Female	15
Pathology performed at 24 months	Control	6 months	Male	32
			Female	16
	Intermittent		Male	32
			Female	16
	Continuous		Male	32
			Female	16
Lifespan	Control	6 months	Male	52
			Female	52
	Intermittent		Male	52
			Female	51
	Continuous		Male	51
			Female	51

**Table 2:** Summary of significantly regulated phenotypes due to the corresponding rapamycin feeding and whether intermittent dosing achieved the level of continuous treatment.

	Phenotype	Intermittent vs control	Continuous vs control	Intermittent vs continuous
**Metabolic**	Glucose tolerance	Reduced	Reduced	Improved
	Body weight	Decreased	Decreased	Increased
	Life span	Increased	Increased	Not significant
**Survival**	Motor coordination	Improved (males)	Improved	Not significant
**Fitness**	Motor activity	Not significant	Improved (males)	Not significant
	Heart rate	Improved	Improved	Not significant
	QT interval	Improved	Improved	Not significant
	Heart hypertrophy	Not significant	Improved (males)	Reduced (males)
	Liver tumour score	Not significant	Improved	Not significant
	Chronic progressive nephropathy	Improved (males)	Improved (males)	Reduced (males)
**Organ pathology**	BAT inflammation	Not significant	Improved (females)	Not significant
	Pancreas inflammation	Not significant	Improved (females)	Reduced (females)
	Kidney inflammation	Not significant	Improved	Reduced
	WAT inflammation	Not significant	Improved	Reduced
	Spleen pathology	Improved (males)	Improved	Not significant

All mouse procedures were conducted in accordance with European, national and institutional guidelines and were approved by the local government authority (Landesamt fur Natur, Umwelt und Verbraucherschutz Nordrhein-Westfalen, Germany) under approval number 84-02.04.2017.A074 and 81-02.04.2019.A313.

## 7 Method details

### 7.1 Metabolic phenotyping

Metabolic phenotyping was performed on mice after six weeks of rapamycin treatment. Rapamycin treatment was started at 10 months of age with 10 mice per treatment in the control and continuous groups. In order to be able to evaluate the effects of intermittent rapamycin feeding after a week with (inter-on) or a week without (inter-off) rapamycin in the diet, the number of animals in this group was doubled to 20 females and males divided into two groups of 10, with administration of rapamycin out of phase by one week in the two groups. Mice that had just ended a week on or a week off rapamycin were thus available simultaneously. After six weeks of rapamycin feeding, glucose tolerance was measured (GTT) and after seven weeks of rapamycin feeding insulin tolerance was tested (ITT). Mice were sacrificed after eight weeks of rapamycin feeding and tissues collected for rapamycin quantification.

For the GTT mice were fasted for 16 h. Body weight and fasted blood glucose levels were measured. Then mice were intraperitoneally injected with 20% glucose (DeltaSelect, solution for infusion, 10 ml/kg body weight). Blood glucose levels were determined 15, 30, 60 and 120 min after glucose injection by making a small incision at the edge of the mouse tail and administering a drop of blood onto a glucose monitor (Accu-Check Aviva, Roche).

For ITT, body weight and blood glucose levels were determined. Each mouse received an intraperitoneal injection of 0.75 units of insulin (in 0.9% NaCl) per kg of body weight (10 ml/kg body weight). Blood glucose levels were determined 15, 30 and 60 min after insulin injection by making a small incision at the edge of the mouse tail and administering a drop of blood onto a glucose monitor (Accu-Check Aviva, Roche).

Rapamycin levels in serum (40 μl) or mouse tissues (30 mg) were measured using mass spectrometry. Tissues were pulverised using a TissueLyser II (Qiagen) and brought into solution with lysis buffer of 6 M guanidinium chloride, 100 mM Tris–HCl, 10 mM TCEP and 40 mM chloroacetamide, to make a protein concentration of 10–20 mg/ml. After heating at 95 °C for 10 min, the protein lysates were sonicated with Bioruptor Plus (Diagenode) connected with a water cooler for 10 cycles (1 cycle of 30 s on, 30 s off). The protein lysate was centrifuged at 20,000*g* for 20 min. The supernatants were transferred to new tubes. After checking the concentration, 200 μg of protein was diluted 10 times with 20 mM Tris–HCl PH 8.3. 1 μg of trypsin was added to each sample and the proteins were digested at 37 °C overnight. The next day, the tryptic digest was stopped by adding formic acid to a final concentration of 1%. The samples were then centrifuged at 20,000*g* for 10 min to remove possible pellets. The supernatants of protein digests were then cleaned and eluted with the home-made StageTips C18 [86]. Mass spectrometry was done in positive ESI MRM (multi reaction monitoring) mode using an Acquitiy UPLCTM I-class System/XevoTM TQ-S (WatersTM) with MassLynx. TargetLynxTM (WatersTM) was used for absolute quantification. The following settings were used: capillary kv 1.5, desolvation temperature 350 °C, desolvation gas flow 800 L/h, cone 150 L/h, collision gas flow 0.15 ml/min. An Acquity UPLC BEH C18 1.7 μm, 2.1 × 100 mm column from Waters was used at 40 °C. Solvent A was 0.22 μm MilliQ-Water containing 10 mM ammonium acetate (Sigma) + 0.1% acetic acid (Sigma) and solvent B 70% acetonitrile/30% isopropanol (VWR) + 10 mM ammonium acetate (Sigma) + 0.1% acetic acid (Sigma). A gradient from 55% A to 0% in 3 min at a flow rate of 0.35 ml/min and an equilibration step from 3.4 min to 7 min were used. The following MRM transitions were used as quantifier (M + H+) + for Rapamycin 917.65m/z to 850.44m/z with a cone at 4 V and collision energy at 18 V. Everolimus was used as internal standard with quantifier MRM transition 975.65m/z to 908.5 and cone at 32 V and collision gas at 16 V. All compounds were dissolved in DMSO (100 μg/ml). A standard mix was prepared of Rapamycin (2000 ng/ml) with a mixture of the starting condition for the UHPLC-conditions. For all compounds a calibration curve was calculated using concentrations from 10 to 1600 ng/ml for Rapamycin. Internal standard (30 μl) was spiked into each calibration solution. Correlation coefficient: *r* < 0.990; response type: internal standard, the peak integrations were corrected manually, if necessary. Quality control standards of each standard were used during sample analysis and showed between 0.5% and 40% deviation respectively. Blanks after the standards, quality control and sample batch proved to be sufficient.

### 7.2 Survival analysis

Survival under control, intermittent or continuous rapamycin treatment from six months on was assessed from a total of 309 C3B6F1 mice, 155 males and 154 females (Table 1). Mice were monitored daily for health and dead mice were counted. Health status of mice was assessed using a score sheet in accordance with the applicable animal welfare rules and the ethical committee guidelines. If a threshold score was reached, mice were sacrificed and inspected for gross pathological changes.

Kaplan–Meier survival curves were generated using the birth and death dates of each individual mouse. Median lifespan was assessed, and survivorship was analysed using log rank test and Cox proportional hazard analysis. Due to survival curves crossing during late life of intermittent and continuously fed females, we used the restricted mean time lost to perform a model-free contrast between these two groups. Maximum lifespan was assessed using a Wang–Allison test [33].

### 7.3 Fitness phenotyping

Phenotyping was done with 45 male and 45 female mice (Table 1) with rapamycin feeding starting at six months of age. Phenotyping was done at 12–15 months of age (middle-aged) and, using the same animals, again at 20–22 months of age (old age), with the exception of indirect calorimetry which was performed only at 15–16 months of age. Longitudinal phenotyping was done in the following order: open field, rotarod, treadmill followed by electrocardiography (ECG). Mice were given at least one week to recover after an experiment. For the open field and treadmill experiments measurements were done while the intermittently fed animals received control food. Rotarod experiments were carried out under rapamycin and control feeding with no statistically significant difference detected in between both groups. The indirect calorimetry experiments spanned several days. Therefore, to maintain a consistent feeding schedule we performed the experiment in six consecutive batches where we ensured an equal distribution of mice at each stage of feeding to represent all aspects of the rapamycin dose.

Open field was used to measure exploratory drive and anxiety-like behaviour at 12 months for middle age mice as well as 20 months and 22 months for old age males and females respectively. Individual animals were placed in an arena measuring 50 × 50 × 40 cm for 10 min. Location was sensed by infrared sensors and then analysed for activity, speed and occupancy of the centre of the arena, which is used as an indicator of anxiety-related behaviour.

Rotarod analysis was performed to test for differences in motor coordination at 12 months for middle age mice as well as 20 months and 22 months for old age males and females respectively. Mice were placed onto the Rotarod (TSE Systems, type 3375-M5) as it was rotating at a low speed (5 rpm). After starting the measurement, the rate of revolution was continuously increased from 5 rpm to 40 rpm over a total period of 300 s. We measured the length of time that each mouse spent on the rod, with a cut-off time of 300 s. The test was performed two consecutive times for each animal and on four consecutive days, with an inter-trial interval of three-four hours.

Treadmill exercise was performed to test for differences in the endurance of mice at 13 months for middle age mice as well as 21 months and 23 months for old age males and females respectively. Mice were placed onto the stationary belt (TSE Systems, type 3033401-M-04/C) and allowed to acclimatise to the new environment for 5 min. The measurement was then started and mice were run to exhaustion, running at a speed of 0.1 m/s for 10 min, before speed was constantly increased to 1.3 m/s within 60 min. To ensure that mice only stopped running upon exhaustion, an electric grid at the end of the belt driven by a light barrier produced a weak stimulus (0.3 mA) as soon as mice slowed down beyond a critical point and crossed the laser beam for more than 2 s. Length of the stimulus was set to 5 s followed by a 5 s refractory period. Exhaustion was defined as the willingness of a mouse to sustain three consecutive shocks instead of returning to the running belt.

Electrocardiography (ECG) was performed and data recorded non-invasively on conscious mice using the ECGenie system (Mouse Specifics: https://mousespecifics.com/heart/ecgenie/) at 13 months for middle age mice as well as 21 months and 23 months for old age males and females respectively. The electrical activity of the heart was recorded via the mouse paws. The ECGenie system is a raised platform enclosed in red Plexiglas. The mouse to be measured was placed freely on the platform on interchangeable electrodes that transmit the electrical signals to a computer. Mice were acclimatised on a training platform for 5 min before the measurement and were measured until at least 400 spikes were cleanly recorded. The recordings were then averaged.

### 7.4 Body composition (MRI) and indirect calorimetry (metabolic cage)

Body fat content was measured in awake mice prior to indirect calorimetry by in vivo nuclear magnetic resonance using a minispec mq 7.5 MHz (Mq7.5 NMR Analyzer, Bruker optics). Awake mice were restrained by placement in a clear plexiglass tube with an inner diameter of 4.5 cm. Measurement time typically did not exceed three minutes.

Indirect calorimetry measurements were done on individual mice using metabolic cages (Phenomaster, TSE Systems) at 15 months of age in males and females. Animals were allowed to acclimatise to the metabolic cages for three to four days before the measurement. Measurements included food and water consumption and spontaneous activity. The difference between the oxygen and carbon dioxide content of the air before and after it was fed into the cages was used to calculate the respiratory quotient. These parameters were measured for two-three days, however only the first 48hrs were used for data analysis. Energy consumption was related to mouse lean mass using the data from the MRI measurement before going into metabolic cages.

### 7.5 Tissue sampling and organ pathology

Tissues for biochemical analysis were harvested after cervical dislocation of the animal. Tissues were rapidly flash-frozen in liquid nitrogen and stored at −80 °C. Tissues for pathology were harvested, fixed in 4% formaldehyde and shipped to Prof. Robert Klopfleisch (Institute of Veterinary Pathology, Freie Universität Berlin) for pathological assessment. Numbers of animals in this cohort (Table 1) were calculated based on obtaining 16 males and 12 females at 24 months of age.

### 7.6 Plasma protein profiling

We quantified 92 plasma proteins from 20 μl of EDTA-plasma by proximity extension assay (PEA) [46] using the Target 96 Mouse Exploratory panel (Olink Proteomics). In brief, an oligonucleotide-labelled antibody probe pair is present for each protein. If they bind to their corresponding target protein in the plasma sample and are in close proximity, then a PCR target sequence is formed. The PCR target sequence is formed by a proximity-dependent DNA polymerisation event, amplified and quantified using real-time PCR, ensuring maximum sensitivity and specificity. Data is normalised within a plate using internal controls in each sample and across plates using inter-plate controls. The values are then set relative to a correction factor determined by Olink®, thus generating the Normalized Protein eXpression (NPX) which is reported on a Log2 scale. A high NPX value corresponds to a high protein concentration and can be linearized by using the formula 2^NPX^. NPX can be used for statistical analysis and express relative quantification between samples but is not an absolute quantification. The Biochemical assays were performed by the company, with the scientists performing the experiment blinded to sample identity. 15 mice per sex and treatment were randomised across plates containing appropriate intra- and inter-plate quality controls from the manufacturer. 3% of samples failed quality control and 1.2% protein measurements were detected as out of range by the manufacturer; low quality samples were removed from further analysis using R4.2.2. One way ANOVA followed by Tukey's post-hoc test was used to compare treatment groups to control mice using “stats” package v4.2.2 [79]. PCA and PLS-DA to evaluate sample clustering and genes contributing to treatment differences were calculated using “mixOmics” package v6.22.0 [80]. Euclidean clustering and heatmaps were generated using “Seaborn” package v0.12.1 on Python v3.9.5 [81]. Enrichment was tested using Fisher's exact test. All characteristics and validation data for each assay are available at the manufacturer's webpage (www.olink.com). Venn diagrams of differentially regulated proteins were made using the web-based tool InteractiVenn [87]. Gene set enrichment and overrepresentation analysis were performed using “clusterprofiler” package v4.6.2 on the list of 92 evaluated proteins [82].

### 7.7 Quantification and statistical analysis

Numbers of mice were estimated to be sufficient to detect statistically meaningful differences of at least 20% between or among groups using standard power calculations with *α* = 0.05 and power of 0.8 on the basis of similar experiments conducted in our laboratory. Homogeneity of variance and normality of residuals were assessed, and appropriate corrections were made if necessary. All experiments were performed in a randomised and blinded fashion when possible. Data were analysed statistically using GraphPad Prism 10.1.0. The value of *α* was 0.05, and data were expressed as ∗*P* < 0.05; ∗∗*P* < 0.01; ∗∗∗*P* < 0.001; ∗∗∗∗*P* < 0.0001 or #*P* < 0.05; ##*P* < 0.01. Spleen size differences were assessed using a Kruskal–Wallis test followed by Dunn's post hoc test. Across group comparisons were made using one-way analysis of variance analysis (ANOVA) and between groups using a Tukey post hoc test. For comparisons of multiple factors (for example, phase ∗ treatment or time∗treatment), two-way ANOVA was reported, followed by Sidak's post-test if interaction between main effects was significant. R 4.2.2 was used for survival statistical analyses using the survival and survRM2 [[83], [84]] packages. Number of animals is reported at the bottom of the bars for each condition or in figure legends. All error bars correspond to standard deviation except for rotarod and longitudinal heart measurements where standard error of the mean is reported as each data point represents the mean of multiple measurements taken from a single animal. Analysis of covariance (ANCOVA) was used for outcome measures with lean mass as a covariate. ANCOVA was plotted with 95% confidence interval bands. Detailed P values for non-significant comparisons, test statistic values, and degrees of freedom are included in Supplementary Table S1.

## CRediT authorship contribution statement

**Maarouf Baghdadi:** Writing – review & editing, Writing – original draft, Validation, Project administration, Methodology, Investigation, Data curation, Conceptualization. **Tobias Nespital:** Project administration, Methodology, Investigation, Data curation, Conceptualization. **Carolina Monzó:** Methodology, Formal analysis, Data curation. **Joris Deelen:** Writing – review & editing, Supervision. **Sebastian Grönke:** Writing – review & editing, Writing – original draft, Supervision, Project administration, Conceptualization. **Linda Partridge:** Writing – review & editing, Writing – original draft, Supervision, Funding acquisition, Conceptualization.

## Declaration of competing interest

The authors declare that they have no known competing financial interests or personal relationships that could have appeared to influence the work reported in this paper.

## Data Availability

Data will be made available on request.
