# Supplementary material for: Intermittent rapamycin feeding recapitulates some effects of continuous treatment while maintaining lifespan extension
Source: Mol Metab. 2024 Feb 13;81:101902. doi: 10.1016/j.molmet.2024.101902 (PMC10900781; doi:10.1016/j.molmet.2024.101902)
Supplement: Multimedia component 1 [file mmc1.docx]

### Supplementary Figure S1 Concentrations of rapamycin in blood and organs

During rapamycin treatment in the intermittent rapamycin regimen, there were comparable rapamycin concentrations to those seen with continuous treatment in liver and WAT of C3B6F1 males (A-D), but a lower concentration in the WAT of C3B6F1 females and brain of both sexes (D-F). In the intermittently treated mice, there was a significant reduction in rapamycin concentration in liver of both sexes and in the WAT of females at the end of the ‘off’ period (A-B, D) but not in the WAT of males or in the brain of either sex (C, E-F). (G) There was no significant difference in rapamycin levels between male and female serum (data from panels Figure 1B). One-way ANOVA followed by Tukey post hoc test was used to calculate statistical significance for all comparisons except for (E) and (F) where Kruskal-Wallis test was used followed by Dunn’s post hoc test. Two-way ANOVA comparing sex and treatment followed by Sidak post hoc test for individual sex comparisons was used for (G). Error bars represent SD. Inter-Off mice were measured after 7 days on control chow, while Inter-On mice were measured after 7 days on rapamycin food. For detailed statistical values see Supplementary Table S1.

### Supplementary Figure S2 Rapamycin treatment does not affect insulin sensitivity

Fasted blood glucose levels in 12 months old (A) male and (B) female C3B6F1 mice. (A) Fasted blood glucose levels were significantly increased in males continuously treated with rapamycin compared to control animals. Intermittent rapamycin feeding also significantly increased blood glucose levels compared to controls, but to a lesser extent than the continuous feeding regime (B). Fasted blood glucose levels in female mice were not significantly changed by rapamycin treatment compared to controls. However, we detected a small but significant reduction of fasted blood glucose in intermittently fed mice currently on rapamycin chow (inter-on) compared to continuously fed females (B). Insulin tolerance test (ITT) of 12 months old (C) male and (D) female mice. Blood glucose levels were plotted against time in minutes after insulin injection. Area under the curve (AUC) analysis of the ITT did not reveal any significant difference in insulin sensitivity in male or female mice treated continuously or intermittently with rapamycin. Number of animals reported at the bottom of the bars. Error bars correspond to SD. Differences between groups were detected using one-way ANOVA followed by Tukey test for multiple comparisons. Detailed statistical values found in Table S1.

### Supplementary Figure 3 Intermittent rapamycin dosing reduced body weight only in males, and to a lesser extent than continuous dosing

Body weight of male (A) and female (B) C3B6F1 mice. Continuous rapamycin treatment produced a substantial reduction in body weight in both sexes, while intermittently treated animals showed a profile more similar to control animals. Body weight was analysed using a mixed-effects model followed by Tukey test for multiple comparison between treatments. *P < 0.05, **P < 0.01, ***P < 0.001, ****P < 0.0001. Error bars indicate SD. For detailed statistical values see Table S1.

### Supplementary Figure 4 Combined survival analysis of C3B6F1female mice from the survival, phenotyping and tissue collection cohort

Kaplan–Meier plot depicting survival of C3B6F1 hybrid female mice belonging to phenotyping, tissue collection and pathology cohorts (Controls n=100, intermittent n=98 and continuous n=98).: *P < 0.05, **P < 0.01, ***P < 0.001, ****P < 0.0001. For detailed statistical values see Table S1. Black arrow indicates the start of the rapamycin treatments, coloured numbers note the median lifespan.

### Supplementary Figure 5 Continuous rapamycin treatment improves motor endurance and exploratory activity in old animals

(A-D) Open field test in middle-aged (A-B) and old (C-D) C3B6F1 mice. Neither intermittent nor continuous rapamycin treatment led to any significant differences in male (A) or female (B) middle-aged (12 months) mice in distance, speed or centre occupancy. (C) Distance and speed were significantly increased in old (20 months) male mice continuously treated with rapamycin compared to control mice, while centre occupancy was not significantly different. (D) No significant difference was detected in distance, speed or centre occupancy between rapamycin treated mice and controls. Number of animals used is denoted at the bottom of each bar. Error bars indicate SD. For detailed statistical values see Table S1.

### Supplementary Figure 6 Rapamycin treatment did not affect energy expenditure or respiratory exchange ratio

Indirect calorimetry was measured in 16 months old male and female C3B6F1 mice. No significant difference was detected in indirect calorimetry adjusted for lean mass of rapamycin treated male and female mice during daytime (A-B) or nighttime (C-D). Respiratory exchange ratio (RER) was not significantly different in rapamycin treated males (E), while a small but significant difference was detected in females (F) during daytime with a similar trend that did not reach significance during nighttime. Number of animals reported at the bottom of the bars. All error bars correspond to SD. For ANCOVA analysis the 95% confidence interval is plotted. Differences between groups were detected using one-way ANOVA followed by Tukey test for multiple comparisons. For detailed statistical values see Table S1.

### Supplementary Figure 7 Rapamycin treatment leads to adverse effects

Histopathological analysis of male and female C3B6F1 mice at 24 months of age for (A) heart, (B) liver (C) kidney and (D) gonad lesions. (A) Intermittent and continuous rapamycin treatment led to significantly increased heart fibrosis in male but no heart fibrosis was observed in females. (B) Intermittent and continuous rapamycin treatment increased liver lipidosis in male and female mice. (C) All male groups suffered from kidney glomerulopathy but no difference was detected among the treatment groups, while females were not severely affected by this pathology.(D) Male gonadal pathology was significantly increased in mice treated with intermittent and continuous rapamycin. Female gonadal pathology was not significantly affected under intermittent or continuous rapamycin treatment. Asterisks denote the following relative to control: *P < 0.05, **P < 0.01, ***P < 0.001, ****P < 0.0001. Hashtag denote the following relative to intermittent rapamycin: # P < 0.05 and ## P < 0.01. For detailed statistical values see Table S1.

##
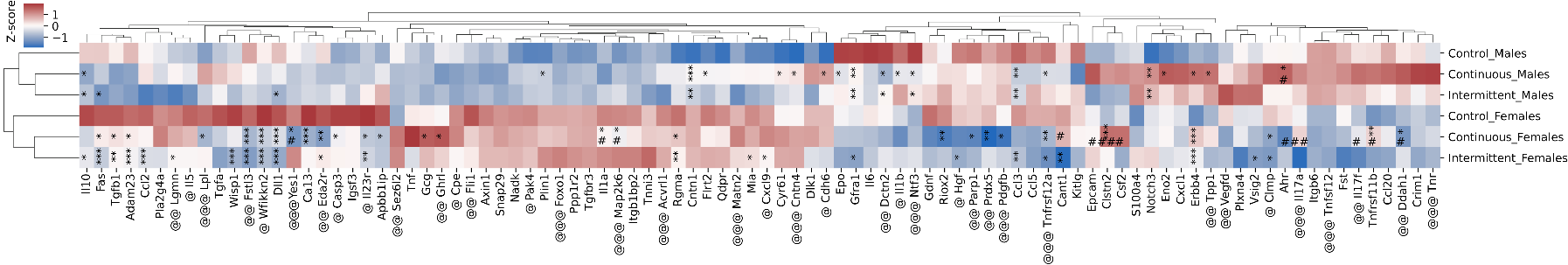
Supplementary Figure 8 Circulating proteins separate in sex- and treatment-specific manner

Circulating proteins were measured using the Olink platform in plasma samples of 24-month-old male and female C3B6F1 mice. Euclidean clustering based on the 92 measured proteins, grouped animals according to males and females, suggesting a sex difference in circulating proteins as well as their response to rapamycin treatment.
